# Preeclampsia, HELLP Syndrome, and Postpartum Renal Failure with Thin Basement Membrane Nephropathy: Case Report and a Brief Review of Postpartum Renal Failure

**DOI:** 10.1155/2020/3198728

**Published:** 2020-11-10

**Authors:** K. C. Janga, Pavani Chitamanni, Shraddha Raghavan, Kamlesh Kumar, Sheldon Greenberg, Kundan Jana

**Affiliations:** Maimonides Medical Center, Brooklyn, NY 11219, USA

## Abstract

A 36-year-old primigravida female from a birthing center was referred for elevated blood pressure to the hospital two days after normal spontaneous vaginal delivery with nausea, vomiting, and diarrhea. During this two-day period, she was experiencing persistent vaginal bleeding and lower abdominal pains for which she took six doses of 600 mg ibuprofen. Further laboratory evaluation reflected leukocytosis, anemia, thrombocytopenia, elevation of liver enzymes, and renal failure with hyperkalemia requiring emergent hemodialysis once in the Medical Intensive Care Unit (MICU). She was diagnosed with HELLP syndrome with underlying preeclampsia. A week later, due to hypertension controlled with medications and nonoliguric renal failure with no active urine sediments, a renal biopsy was indicated to direct management. The renal biopsy supported the diagnosis of diffuse severe acute tubulointerstitial nephritis with hypereosinophilia and thin basement membrane nephropathy (see figures). She was subsequently treated with high-dose steroids which resulted in the normalization of blood pressures and renal function returning to baseline. We report the first case of acute tubulointerstitial nephritis in an individual with thin basement membrane nephropathy secondary to postpartum complications.

## 1. Case Summary

### 1.1. History of Presenting Illness

Our patient is a 36-year-old female primigravida who presented with two-day history of persistent postpartum vaginal bleeding, pelvic pain, nausea, vomiting, diarrhea, and poor oral fluid intake. Patient had received prenatal care limited to vitamins and genetic counselling and went into labor at 41-week-and-4-day gestation at a midwife-assisted birthing center. Castor oil, herbal tea techniques, acupuncture, and various massage maneuvers were used to induce and aid the progression of labor. The patient delivered 11 hours after an artificial rupture of membranes performed by the midwife. Due to a second-degree vaginal tear and postpartum hemorrhage of unknown etiology and duration, oral misoprostol and intramuscular oxytocin were administered, and the patient returned home. Over the next day, she had poor oral intake, pelvic pain, and low urine output. Due to continuing weakness, difficulty walking, and high blood pressure, she was referred to our medical facility. The patient's past medical history includes easy bruising, nosebleeds, and bleeding gums since childhood. Additionally, she used combined hormonal contraceptives for three years prior to her current gestation.

### 1.2. Hospital Course

In the MICU, the patient was in severe renal failure with metabolic acidosis; azotemia; persistent hyperkalemia even after administration of calcium gluconate, insulin dextrose, bicarbonate pushes, and 2 doses of Kayexalate; hyponatremia unresponsive to fluids; and hypoglycemia. Laboratory findings also included elevated liver enzymes, anemia, thrombocytopenia, leukocytosis, hyperuricemia, subnephrotic-range proteinuria with a urine protein to creatinine ratio of 0.77 (770 mg/dl) and high LDH levels suggestive of HELLP syndrome with preeclampsia. The patient was subsequently started on a nicardipine drip for elevated blood pressures. Although presenting with no neurologic signs, she was started on seizure prophylaxis for eclampsia with levetiracetam. The patient was also placed on broad-spectrum antibiotics for possible intra-abdominal infection. Though the patient was afebrile, her abdominal pain with leukocytosis and history of postpartum hemorrhage prompted initiation of prophylactic treatment for puerperal sepsis. Differential diagnoses at that time included preeclampsia/HELLP syndrome, acute fatty liver of pregnancy (AFLP), hemolytic uremic syndrome (HUS), thrombotic thrombocytopenic purpura (TTP), and puerperal sepsis.

Due to persistently elevated creatinine and refractory hyperkalemia of 6.3 mg/dl not responding to medical therapy, the patient underwent one cycle of emergent hemodialysis in the Medical Intensive Care Unit. Though her liver function tests, platelets, hematocrit, and electrolytes began to slowly normalize over the next two days, her creatinine remained elevated. She was diagnosed with nonoliguric renal failure of uncertain etiology requiring a kidney biopsy.

By hospital day 5, the patient's electrolytes, leukocytosis, thrombocytopenia, and hemolysis parameters normalized (Table 1). Seizure and antibiotic prophylaxes were discontinued at that time. Her blood pressure remained elevated, and the patient was deescalated to oral labetalol and amlodipine. ADAMTS13 activity levels, ordered on admission, showed a low value of 36.8% (normal 66.8%). However, TTP was ruled out as ADAMTS13 levels should be under 10% for this diagnosis. Rare schistocytes were seen on daily peripheral blood smears; thus, the patient still partially met criteria for atypical HUS. Immunologic workup and viral panels for hepatitis and HIV were unremarkable, with the exception of Epstein-Barr virus immunoglobulin titers evident for chronic infection.

**Figure 1:**
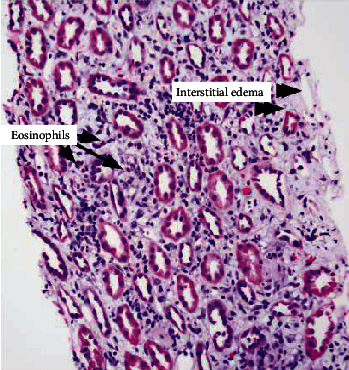
Light microscopy findings indicative of diffuse severe acute tubulointerstitial nephritis with eosinophils.

**Figure 2:**
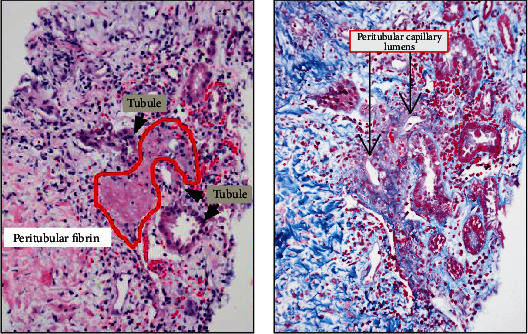
(a, b) Light microscopy shows fibrinoid necrosis of the peritubular areas suggestive of microangiopathy.

**Figure 3:**
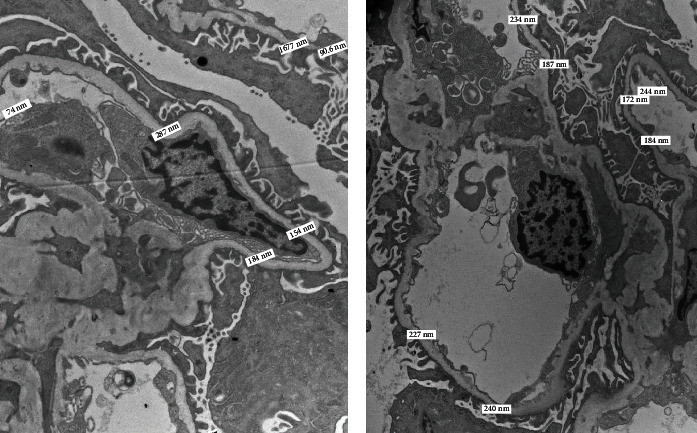
(a, b) Electron microscopy demonstrates diffuse glomerular basement thinning with arithmetic mean 215.06 nm (range 135-287 nm with 100% of 41 measures less than 300 nm and 63% less than 215 nm).

On hospital day 6, the patient regained normal appetite and she was participating in breastfeeding and out-of-bed exercises conducted in the medical ward. Renal biopsy results demonstrated acute tubulointerstitial nephritis with eosinophils and thin glomerular basement membrane (Figures 1–3). Thus, the patient was started on high-dose steroids with a rapid taper over 2 weeks. The management we provided resulted in normalization of renal function.

## 2. Renal Biopsy Results

## 3. Discussion

The syndrome of postpartum renal failure has been recognized since 1968 as an idiopathic condition characterized by renal failure in association with microangiopathic hemolytic anemia and thrombocytopenia occurring within a week of apparently normal delivery [1, 2] Postpartum renal failure is associated with high mortality and morbidity primarily from bleeding diathesis and renal failure. Beyond supportive care and hemodialysis, there is no conclusive evidence that specific therapy is effective in mitigating the hemolytic abnormality or reducing the severity of renal failure. Postpartum renal failure can be categorized based on etiologies similar to the nonpregnant population (Table 2).

## 4. Thin Basement Membrane Nephropathy and Pregnancy

Thin basement membrane nephropathy (TBMN) is characterized by persistent hematuria and thinning of glomerular basement membrane. This disease is clinically defined as isolated glomerular hematuria, minimal proteinuria, normal renal function, and a first-degree relative with hematuria. It is histologically defined by uniform basement membrane thinning (on electron microscopic examination) in the absence of glomerular or interstitial abnormality (on light microscopic or immunological examination). Additionally, WHO criteria for defining TBMN is a basement membrane dimension between 250 and 264 nm [3]. Causes of TBMN include focal segmental glomerulosclerosis (FSGS) and IgA glomerulosclerosis; however, many patients have incidental biopsy findings as with our patient.

Clinical complications of TBMN include hematuria, hypertension, proteinuria, and renal failure [4]. Out of these, hypertension and hematuria are the most common complications. However, hypertension in TBMN is not known to be causal or coincidental. Renal failure is a rare complication of TBMN and is usually known to be precipitated when associated with proteinuria, hypertension, and a coexistent tubulointerstitial or glomerular lesion as seen in our patient [5]. Renal impairment in TBMN is explained by progression of the disease itself or more commonly, predisposition to glomerular or tubulointerstitial lesion [6, 7]. In a study of 16 biopsy-proven TBMN patients with acute renal failure, 15 of them had coexisting glomerular or tubulointerstitial lesions suggesting the prominent role they play in leading to renal failure [5]. Whereas lesions like acute tubulointerstitial nephritis, as seen in our patient, are more likely to be coincidental, lesions like IgA glomerulonephritis need further investigation to understand their association.

Although the disease affects >1% of the population, the effects of TBMN have not been extensively studied in pregnancy. In a study from 2005, records of parous women with a diagnosis of TBMN over 13 years were reviewed. Majority of the patients (89.6%) had no complications with an overall fetal outcome of 95% live births and 4.4% fetal loss in <12 weeks. Particularly, 3 percent of them developed renal impairment, 48 percent developed hypertension, and 53 percent developed proteinuria. Renal impairment was reversible in 100 percent of cases [3].

Our patient presented with nonoliguric acute renal failure with hyperkalemia postpartum warranting a session of emergent hemodialysis. Renal biopsy showed ultrastructural diffuse glomerular basement membrane thinning (mean 215.06 nm; range 135-287 nm) consistent with thin glomerular basement membrane nephropathy. Although patients with thin basement membrane nephropathy are known to have an overall benign course, patients developing proteinuria or hypertension tend to have a higher risk of renal failure [8]. Proteinuria and hypertension in our patient were also associated with elevated liver enzymes and thrombocytopenia suggestive of a broad differential including preeclampsia, HELLP syndrome, acute fatty liver of pregnancy, and atypical hemolytic uremic syndrome.

In addition, biopsy finding of acute tubulointerstitial nephritis with hypereosinophilia also suggested acute interstitial nephritis (AIN). Overall, the etiology of acute interstitial nephritis can be grouped into five general categories: drug-induced, infectious, autoimmune, malignant, and idiopathic. Out of these, drug-induced AIN accounts for over two-thirds of all cases and, although many drugs have been implicated, nonsteroidal anti-inflammatory drugs (NSAIDs) and antimicrobial agents have been seen to cause the majority of these cases. It is understood that the pathogenesis of AIN is an immunologic reaction via endogenous nephritogenic or exogenous antigens that are presented by tubular cells to T cells, activating a cascade of effector cells and cytokines eventually leading to interstitial infiltrates [9]. Either patchy or diffuse infiltrates of lymphocytes, macrophages, and plasma cells are commonly seen on renal biopsy, such as in our patient, along with nonsuppurative fluid within the interstitium and/or tubules. The laboratory findings, showing impairment in kidney function, can vary from selective abnormalities of tubular function to acute renal failure with or without oliguria. Historically, BUN and creatinine will increase after tubular dysfunction is detectable, with or without oliguria. The presence of a history consistent with AIN and laboratory findings of acute kidney injury are sufficient to diagnose this condition, although definitive diagnosis is through renal biopsy. Reversal of kidney injury is the rule in 60-65% of cases after the removal of offending agents. The use of steroids in AIN remains controversial but deserves consideration, as it has been shown to have better outcomes for patients when started early in the disease course [9, 10]. The etiology of AIN in our patient was likely the recent use of NSAIDs prior to delivery.

## 5. Differential Diagnoses

At presentation, the patient met the criteria for preeclampsia with hypertension, proteinuria with urine protein to creatinine ratio of 0.77, elevated creatinine and transaminases, and thrombocytopenia suggestive of end-organ damage (Table 3). Currently, preeclampsia is one of the most prevalent medical complications of acute kidney injury in women and the most reported complication in those women requiring dialysis in the postpartum period [11].

The pathogenesis of preeclampsia occurs in several ways. It involves defects in placentation mediated by placental-derived angiogenic factors, such as placental growth factor or soluble endoglin. Along with dysfunction of the complement system, errors in placentation can cause severe kidney injury. Without maternal susceptibility, the complement system is activated in normal pregnancy. However, it is exaggerated in preeclampsia and studies have found this exacerbation cytotoxic to cells [12]. It has also been found that preeclampsia has a direct effect via endothelial injury, vasoconstriction, inflammation, and the overactivation of the coagulation cascade. These mechanisms are sufficient enough to cause catastrophic organ complications and are responsible for high admission rates to the intensive care unit.

HELLP syndrome was also part of the differential as the patient had evidence of hemolysis with elevated liver enzymes and low platelet count (Table 4). This syndrome can present up to 7-day postpartum and is a milder form of microangiopathic hemolytic anemia due to a dysregulation of the complement pathway. Postpartum complications involve bleeding requiring transfusions, DIC, pulmonary edema, and renal failure in 10% of recorded cases. Management is usually supported including magnesium sulfate, antihypertensive medications, and blood transfusions for Hb < 7, and platelet transfusions for <30K. This syndrome is self-limiting in the postpartum phase, and trending the platelet counts is used as a prognostic indicator [13, 14].

Though acute fatty liver of pregnancy (AFLP) is known to typically present between the 30^th^ and 38^th^ week of pregnancy, few cases have been reported to occur postpartum [15]. General clinical symptoms that can be present at the time of diagnosis are nausea, vomiting, abdominal pain, and jaundice. The pathogenesis of this disease involves maternal and fetal mutations in the enzyme long-chain 3-hydroxyacyl-CoA dehydrogenase, which processes long-chain fatty acids (LCFA). With dysfunction of this enzyme, LCFAs accumulate and cause hepatotoxicity. Thus, although biopsy is not usually performed, it would demonstrate microvesicular fatty infiltration of hepatocytes [12]. Pregnancy-related acute kidney injury is present in over half the cases of AFLP, as these LCFAs can deposit in the renal tubular epithelium. Our patient similarly reported nausea and vomiting in the postpartum period. A large degree of clinical overlap is very often seen between AFLP, HELLP, and preeclampsia [16]. With elevated transaminases, hypoglycemia, lactic acidosis, and thrombocytopenia, she fell into the spectrum of this disease, although her hypertension also placed her diagnosis in the realm of preeclampsia/HELLP syndrome. Since distinguishing between AFLP and preeclampsia/HELLP can be difficult due to similar parameters of disease, many patients, about 20-40% with AFLP, have a simultaneous diagnosis of preeclampsia/HELLP [12].

Thrombotic microangiopathies were also considered an important differential as the patient had hemolytic anemia, acute kidney injury (AKI), and thrombocytopenia. However, thrombotic thrombocytopenic purpura was ruled out as ADAMTS13 levels were >10%. Atypical hemolytic uremic syndrome (HUS) was still a possibility with marked effect on renal function requiring renal replacement therapy. In the postpartum period, loss of placental protective barrier mechanisms, inflammation, infection, wounded uterus, and influx on fetal cells can trigger systemic activation of alternative complement cascade. Genetic mutations in the regulatory proteins (factor-H, I, CD-46) or complement activators (C3, factor-B) of the alternative complement pathway are found in 50% of the cases [13, 17, 18].

Preeclampsia, AFLP, and HELLP syndrome are commonly associated with cortical or acute tubular necrosis on renal biopsy [19] whereas atypical HUS shows fibrinoid necrosis of arterioles and thrombus in the lumen extending to the glomerular capillary tufts [18]. However, renal biopsy in our patient showed acute tubulointerstitial nephritis with hypereosinophilia and thinning of the basement membrane suggestive of acute interstitial nephritis with thin basement membrane nephropathy. A rare focus of microscopic peritubular fibrin and necrosis was also present (Figure 2(a)) suggestive of a microangiopathy; however, other features of thrombotic microangiopathy (e.g., arterial, arteriolar, or glomerular angiopathy/ultrastructural endotheliosis) were not identified.

In conclusion, we present a case of renal failure in the postpartum period in which an array of factors were considered. Predisposing risk factors for postpartum renal failure include nephrotoxic medications, infections, proteinuria, cystic diseases, chronic kidney disease, transplant, and glomerular diseases. As outlined by our review article, a few of these causes include medication use like NSAIDS in the peripartum period and gestational disease including preeclampsia and HELLP syndrome, as well puerperal sepsis in the postpartum obstetrical infections. Our patient was successfully treated with prompt supportive therapy and a short course of high-dose steroids with normalization of renal indices and urine proteinuria. In such cases of renal failure specifically in the postpartum period, the etiologies contributing are overlapping each other leading to a complex presentation which requires a multidisciplinary approach in the treatment geared towards addressing each of the specific causes [20].

## Figures and Tables

**Table 1:** Laboratory values of the patient over the course of her hospitalization.

	Day 1	Day 3	Day 5	Day 7	Discharge	3-week follow-up
Glucose	40	147	73	109	96	83
Hemoglobin	8.9	8.5	7.9	7.4	7.0	11.4
WBC count	23.7	16.6	9.1	8.0	8.6	6.4
Eosinophils (%)	0.0	0.0	1.1	1.6	0.0	
Platelet count	57000	115000	144000	183000	253000	211000
Creatinine	5.3	4.7	5.6	5.3	2.1	1.0
BUN	57	45	61	66	53	17
AST	650	166	45	32	18	13
ALT	1308	708	313	184	91	15
ALP	143	142	119	111	102	69
Sodium	113	126	123	132	142	140
Potassium	6.6	4.4	4.2	4.6	4.8	4.0
Bicarbonate	17	20	19	19	21	30
Bilirubin	0.8	0.5	0.4	0.4	0.4	0.4
PT	10.2	11.2		11		
PTT	26.5			30.3		
INR	0.9	0.9		0.9		
LDH	448	360		278	224	

WBC: white blood cell; BUN: blood urea nitrogen; AST: aspartate transaminase; ALT: alanine transaminase; ALP: alkaline phosphatase; PT: prothrombin time; PTT: activated partial thromboplastin time; INR: international normalized ratio; LDH: lactate dehydrogenase.

**Table 2:** Causes of renal failure in pregnant and nonpregnant patients.

Etiologies of renal failure	Conditions related to pregnancy	Conditions unrelated to pregnancy
Prerenal	Hyperemesis gravidarum, vomiting due to preeclampsia, HELLP/AFLP, hemorrhage (missed abortion, septic abortion, placental abruption, placenta previa, uterine previa, bleeding during surgery, uterine laceration, uterine perforation)	Vomiting due to infections, gastroenteritis, pyelonephritis, malnutrition Others (like diuretics, CHF)
Intrinsic renal	ATN/ACN: preeclampsia, HELLP, AFLP, amniotic fluid embolism, pulmonary embolism TMA: HUS, preeclampsia, HELLP, AFLP, DIC, underlying glomerular diseases	ATN, de novo glomerular disease, AIN
Postrenal	Bilateral hydronephrosis Trauma to the ureters and bladder during surgery	Bilateral ureteral obstruction stone/tumor Tubular obstruction (meds/calcium/uric acid) Obstruction at bladder outlet
Renal allograft related	Similar to nonpregnant conditions	Acute rejection ATN Acute interstitial nephritis, calcineurin inhibitor toxicity, CMV/BK virus nephropathy, postinfectious glomerulonephropathy

HELLP: hemolysis, elevated liver enzymes, low platelet count; AFLP: acute fatty liver of pregnancy; CHF: congestive heart failure; ATN: acute tubular necrosis; ACN: acute cortical necrosis; AIN: acute interstitial nephritis; TMA: thrombotic microangiopathies; HUS: hemolytic uremic syndrome; DIC: disseminated intravascular coagulation; CMV: cytomegalovirus.

**Table 3:** Diagnostic criteria for preeclampsia fulfilled by our patient.

	Preeclampsia criteria	Our patient's laboratory results on admission	Met or unmet
Blood pressure	New-onset hypertension after 20 weeks of gestation (>140/>90 on 2 occasions at least 4 h apart or >160/>110 within short minutes)	Postpartum blood pressure of 140/60 mmHg, with continuous elevated readings during hospitalization	Met
Proteinuria	Hypertension plus >300 mg/24 h urine collection, protein/creatinine ratio of >0.3, or urine dipstick reading of 1+	Protein/creatinine ratio of 0.7	Met
End-organ damage	Blood pressure criteria plus evidence of thrombocytopenia (platelet < 100K), renal insufficiency (creatinine > 1.1 or doubling), impaired liver function (transaminases twice the normal), pulmonary edema, cerebral or visual symptoms	Platelets: 68,000/*μ*l	Met
		Creatinine: 5.3 mg/dl	
		AST/ALT: 650/1308	

AST: aspartate transaminase; ALT: alanine transaminase.

**Table 4:** Diagnostic criteria for HELLP syndrome fulfilled by our patient.

Definition	Laboratory range	Met or unmet
Hemolysis	Peripheral smear: schistocytes and burr cells (>5/HPF)	Yes
	Serum bilirubin: >1.2 mg/dl	No
	Haptoglobin: <25 mg/dl	Yes
	LDH > 2x the upper limit of normal	Yes
	Anemia: Hgb < 7 and not related to blood loss	Yes
Elevated liver function tests	AST/ALT > 2x the upper limit of normal	Yes
Low platelets	<100,000 cells/*μ*l	Yes

AST: aspartate transaminase; ALT: alanine transaminase.

## Data Availability

All data generated or analyzed during this study are included in this published article.
